# Exercise-Induced Plasma Surfactant Protein B Response in Advanced Heart Failure: Relation to Exercise Limitation, Resting Invasive Hemodynamics, and Clinical Outcomes

**DOI:** 10.3390/ijms27146461

**Published:** 2026-07-21

**Authors:** Anna Drohomirecka, Katarzyna Kozar-Kamińska, Joanna Waś, Anna Rochon, Anna Kasprzyk-Pawelec, Tomasz Zieliński, Tomasz Rywik

**Affiliations:** 1 National Institute of Cardiology, Alpejska 42, 04-628 Warsaw, Polandjwas@ikard.pl (J.W.); ak1801@georgetown.edu (A.K.-P.); tzielinski@ikard.pl (T.Z.); trywik@ikard.pl (T.R.); 2 Department of Biochemistry and Molecular & Cellular Biology, Georgetown University, 37th and O Streets, Washington, DC 20057, USA

**Keywords:** pulmonary vascular stress, alveolar–capillary barrier, surfactant B, heart failure, exercise intolerance

## Abstract

Plasma surfactant protein B (SPB) is a marker of alveolar–capillary barrier injury and is elevated in heart failure (HF). We assessed whether cardiopulmonary exercise testing (CPET) changes plasma SPB in advanced HF and whether the SPB response relates to exercise limitations, resting pulmonary hemodynamics, and outcomes. Fifty-one patients (mean age 55.6 ± 7.5 years) with advanced HF (NYHA II–III, left ventricular ejection fraction ≤ 35%) underwent comprehensive evaluation, including CPET and invasive hemodynamic assessment. Blood samples were collected before and after CPET. Patients were followed for 23.5 ± 12.6 months. The composite endpoint included death, urgent heart transplantation, or left ventricular assist device implantation. SPB concentrations increased after CPET (median [IQR]: 77.5 [60.5–106.5] vs. 87.2 [63.5–111.9] ng/mL; *p* < 0.001). Greater relative increases occurred in NYHA III patients and correlated with NT-proBNP. Relative SPB changes correlated positively with the VE/VCO_2_ slope and negatively with the anaerobic threshold but not with resting systolic pulmonary artery pressure or pulmonary vascular resistance. Neither baseline nor post-exercise levels, nor their changes, predicted clinical outcomes. In conclusion, the SPB response to exercise reflects dynamic pulmonary barrier stress related to functional limitations in advanced HF but lacks prognostic value in this cohort.

## 1. Introduction

The alveolar–capillary barrier, also referred to as the air–blood barrier, is created by the pulmonary microvascular endothelium and alveolar epithelium [1]. Its primary function is to facilitate efficient gas exchange during the breathing process while simultaneously preventing the leakage of fluid and macromolecules from the capillary vessels into the alveolar space [1,2]. Under physiological conditions, the alveolar capillary membrane is covered by a pulmonary surfactant—a lipid-rich film synthesized by alveolar type II epithelial cells. It is composed mainly of phospholipids (about 80%), followed by neutral lipids; the two hydrophobic peptides (1–2%), surfactant protein B (SPB) and surfactant protein C (SPC) [2]; and the hydrophilic proteins, surfactant protein A and D (~5–6%) [3]. The surfactant is responsible for lowering intra-alveolar surface tension and preventing alveolar collapse, while SPB was revealed to particularly achieve and sustain the lowest tensions during compression of the surface film during expiration [2]. Components of the surfactant, such as SPB, may leak into the bloodstream in pathological conditions involving alveolar–capillary barrier disruption—for example, in acute or chronic lung injury, heart failure, or mechanical ventilation-induced stress [1,4].

Heart failure (HF) is characterized by an increase in left ventricular end-diastolic pressure and, as a consequence, by pulmonary capillary stasis [5]. Elevated capillary pressure affects the alveolar–capillary membrane, which may lead to structural disruption, increased permeability to water and ions, and impairment of local regulatory mechanisms essential for normal gas exchange [5]. Moreover, as shown by De Pasquale et al. [6], acute stress failure—such as cardiogenic pulmonary edema—results in a prolonged leakage of surfactant proteins into the circulation, even after hemodynamic abnormalities have resolved. The authors suggested that signs of prolonged alveolar–capillary barrier damage may be related to pulmonary parenchymal inflammation. In chronic HF, structural and functional remodeling of the alveolar–capillary barrier has also been observed. However, these changes involve additional pathophysiological processes, such as interstitial fibrosis, thickening of the basement membrane, increased extracellular matrix deposition (particularly collagen type IV), and reduced efficiency of fluid clearance mechanisms [5].

A few studies have reported the association between the levels of circulating surfactant proteins and prognosis in HF, beginning with the pioneering study of Pasquale et al. [7], who demonstrated that SPB not only reflected the clinical status of HF patients (plasma levels of SPB were higher than in healthy controls and increased with NYHA class or demand on diuretics) but also was independently associated with HF hospitalizations. Then, Magri et al. [8] confirmed the prognostic relevance of surfactant proteins; however, they demonstrated that only the elevated immature form of SPB, but not the mature one, was independently associated with a higher risk of hospitalization in patients with stable chronic HF.

Against this background, the present study was designed to evaluate whether physical exertion induces an increase in circulating surfactant protein B levels in patients with HF and to determine whether the potential exercise-related response carries any prognostic significance.

## 2. Results

**Figure 1:**
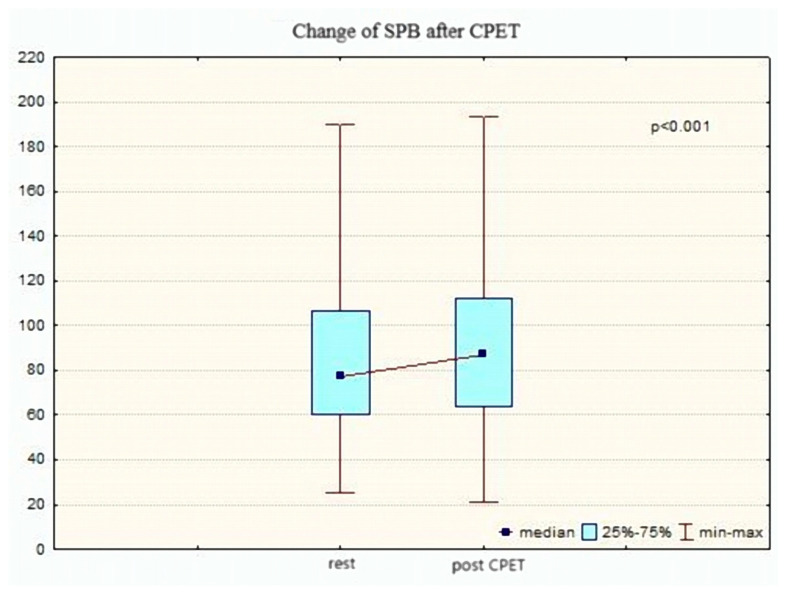
Plasma concentrations of surfactant B at rest and after cardiopulmonary exercise test. CPET—Cardiopulmonary exercise test; SPB—surfactant B.

Plasma SPB concentrations increased significantly after cardiopulmonary exercise testing (CPET) compared with resting values (median [IQR]: 87.2 [63.5–111.9] vs. 77.5 [60.5–106.5] ng/mL; *p* < 0.001) (Figure 1, Supplementary Figure S1A,B).

The median relative change in SPB concentration after exercise, adjusted for baseline levels, was 5.3% [IQR −1.9 to 13.3%].

Higher SPB concentrations were associated with more advanced HF. NT-proBNP levels correlated with both resting SPB (R_S_ = 0.32, *p* = 0.02) and post-exercise SPB concentrations (R_S_ = 0.41, *p* = 0.003). In addition, the relative exercise-induced change in SPB was positively correlated with NT-proBNP (R_S_ = 0.38, *p* = 0.007) (Supplementary Figure S2).

Patients with worse functional status exhibited a greater increase in SPB levels after exercise. The relative increase in SPB was significantly higher in patients with more advanced symptoms of HF (NYHA class III) compared with those in NYHA class II (median 8.7% [IQR 2.4–16.8%] vs. 1.16% [IQR −3.0–8.6%], *p* = 0.02).

The exercise-related increase in SPB was also associated with reduced exercise capacity. A higher post-exercise SPB change correlated positively with the VE/VCO_2_ slope (R_S_ = 0.29, *p* = 0.04) and inversely with the anaerobic threshold (R_S_ = −0.44, *p* = 0.002).

SPB concentrations measured at rest, after CPET, and their exercise-induced change did not show significant correlations with invasive pulmonary hemodynamic parameters. Neither systolic pulmonary artery pressure nor pulmonary vascular resistance was associated with circulating SPB levels (Table 1).

Additional exploratory analyses showed that resting SPB, post-exercise SPB, and the relative exercise-induced SPB change did not differ significantly between men and women (*p* = 0.21, *p* = 0.12, *p* = 0.68) or between patients with ischemic and non-ischemic HF etiology (*p* = 0.49, *p* = 0.93, *p* = 0.85).

In addition, age was not significantly correlated with resting SPB (R_S_ = 0.12, *p* = 0.41), post-exercise SPB (R_S_ = 0.12, *p* = 0.40), or relative SPB change (R_S_ = −0.07, *p* = 0.61).

Furthermore, no significant associations were found between medical therapy and SPB-related variables. Resting SPB, post-exercise SPB, and the relative exercise-induced SPB change did not differ according to beta-blocker selectivity (beta1-selective vs. non-selective; *p* = 0.95, *p* = 0.89, and *p* = 0.34, respectively) or the achievement of the target beta-blocker dose (*p* = 0.10, *p* = 0.13, and *p* = 0.99, respectively). Similarly, peak VO_2_, percentage of predicted peak VO_2_, anaerobic threshold, and VE/VCO_2_ slope did not differ according to beta-blocker selectivity or achievement of the target beta-blocker dose (all *p* > 0.05). Sildenafil treatment was also not associated with resting SPB, post-exercise SPB, or relative SPB changes (*p* = 0.31, *p* = 0.17, and *p* = 0.72, respectively), nor with the CPET-derived parameters listed above (all *p* > 0.05).

**Figure 2:**
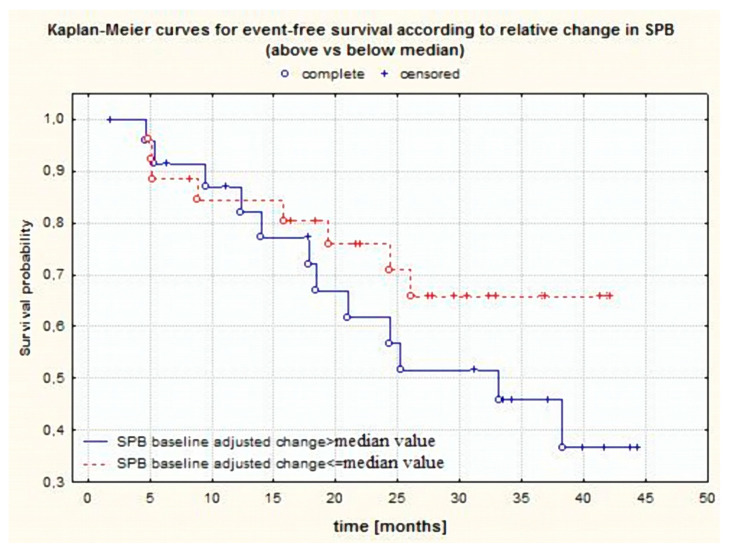
Probability of composite event-free survival according to the relative change in plasma surfactant B concentration after cardiopulmonary exercise testing. SPB—Surfactant B.

The mean follow-up duration was 23.5 ± 12.6 months, ranging from 2 to 44 months for all patients. It ranged from 2 to 38 months for patients whose observation was censored (due to heart transplantation or LVAD implantation, regardless of urgency) or who reached the study’s endpoint (death, urgent heart transplantation, or urgent LVAD implantation, whichever occurred first), and it ranged from 22 to 44 months for the remaining patients. During the observation period, 14 patients died, and a total of 20 composite endpoints occurred. An additional nine patients were censored at the time of non-urgent heart transplantation or LVAD implantation. In Cox proportional hazards analyses, neither baseline SPB (HR = 1.00, 95% CI: 0.98–1.01), post-exercise SPB (HR = 1.00, 95% CI: 0.99–1.01), nor the relative change in SPB after CPET (HR = 2.98, 95% CI: 0.25–35.2) was associated with clinical outcomes (composite endpoint). Kaplan–Meier analysis stratified according to the median relative change in SPB and the results of the log-rank test (χ^2^ = 1.17, *p* = 0.24) showed no significant difference in event-free survival between groups (Figure 2). In Cox proportional hazards analyses, neither baseline SPB, post-exercise SPB, nor the relative change in SPB after CPET was associated with overall survival (HR = 1.00, 95% CI: 0.98–1.01; HR = 1.00, 95% CI: 0.99–1.01; and HR = 6.36, 95% CI: 0.39–102.4, respectively). Kaplan–Meier analysis stratified by the median relative SPB change likewise showed no significant difference in survival between groups (log-rank χ^2^ = 1.29, *p* = 0.20).

## 3. Discussion

Acute exercise induces a complex systemic response that involves multiple organs and regulatory pathways. Beyond the well-recognized hemodynamic and metabolic adaptations, a single bout of exercise leads to broad changes in the circulating plasma milieu, including alterations in proteins, metabolites, and nucleic acids [9]. Numerous tissues release signaling molecules into the bloodstream during exertion, and these exercise-responsive factors—collectively referred to as exerkines—mediate interorgan communication through autocrine, paracrine, and endocrine mechanisms. Consequently, acute exercise is associated with measurable changes in the plasma proteome and metabolome, reflecting activation of diverse biological pathways.

Previous investigations from our group have also demonstrated that exercise induces significant biochemical changes in the circulation of patients with HF [10,11]. In earlier studies, cardiopulmonary exercise testing was associated with alterations in metabolites related to nitric oxide metabolism, suggesting a dynamic modulation of endothelial and vascular function during exertion [10]. Moreover, proteomic analyses performed in a smaller cohort revealed that a single bout of exercise modifies the expression of a substantial number of circulating proteins, many of which are involved in immune regulation, inflammatory pathways, coagulation, and cellular stress responses [11]. These findings underscore that the systemic response to physical exertion in HF is complex and involves multiple interconnected biological systems.

Among the organ systems affected by exercise, pulmonary circulation represents a particularly relevant component of the cardiopulmonary interaction in HF. Increased pulmonary microvascular pressure during exertion may impose mechanical stress on the alveolar–capillary membrane, potentially leading to transient alterations in its permeability. In this context, surfactant protein B (SPB) may provide a unique insight into pulmonary responses to exercise, as it is synthesized exclusively by type II pneumocytes and has no known extrapulmonary source [3]. Therefore, circulating SPB levels may serve as a specific marker of the dynamic changes occurring within the alveolar–capillary unit during exercise in patients with HF.

Elevated pulmonary microvascular pressure has been shown to disrupt the alveolar–capillary membrane and promote leakage of surfactant components into the circulation when barrier integrity is compromised [6,12]. A similar mechanism was described in studies analyzing alveolar–capillary membrane dysfunction in chronic HF, including the work by Banfi et al. [1], which demonstrated that long-term elevation of pulmonary capillary pressure may lead to structural damage and increased permeability of the barrier. This interpretation is further supported by more recent evidence showing that immature SPB is also elevated in patients with severe calcific aortic stenosis, a condition likewise associated with chronic pulmonary venous hypertension and alveolar–capillary membrane dysfunction, suggesting that surfactant leakage may represent a broader marker of chronic hemodynamic lung injury in left-sided heart disease rather than a phenomenon restricted to HF alone [13]. In this context, a single bout of intense exercise, such as cardiopulmonary exercise testing, may represent an acute hemodynamic challenge superimposed on the chronic structural alterations of the alveolar–capillary unit present in HF. Consequently, the observed increase in circulating SPB after exercise may reflect both transient pulmonary vascular stress induced by exertion and the underlying vulnerability of the alveolar–capillary membrane resulting from chronic HF-related remodeling.

In line with this pathophysiological framework, we observed a significant rise in circulating SPB concentrations following cardiopulmonary exercise testing. Moreover, the magnitude of the exercise-induced increase was greater in patients with more advanced functional impairment and was related to established indices of ventilatory inefficiency, including the VE/VCO_2_ slope and anaerobic threshold. These findings support the hypothesis that physical exertion may transiently exacerbate alveolar–capillary barrier dysfunction in patients with HF. This interpretation is further supported by the study of Magrì et al. [14], who showed that circulating plasma SPB levels in chronic HF were related not only to impaired alveolar gas diffusion, particularly membrane diffusion capacity, but also to peak VO_2_ and the VE/VCO_2_ slope, thereby linking SPB with both pulmonary membrane dysfunction and reduced exercise performance. The association between SPB dynamics and reduced exercise capacity is also physiologically plausible. HF is characterized by chronic pulmonary congestion. Consequently, it may lead to abnormalities in pulmonary gas exchange resulting from structural changes within the alveolar–capillary unit, including interstitial fibrosis, reduced diffusion capacity, and remodeling of the pulmonary microvasculature [5,15]. These alterations contribute to impaired ventilatory efficiency and exercise intolerance, which are hallmark features of the syndrome. In this context, circulating markers reflecting alveolar–capillary barrier dysfunction may represent a biochemical correlate of the pulmonary component of exercise limitations in HF.

Moreover, in the present study, SPB concentrations correlated with NT-proBNP levels both at rest and after exercise, suggesting a relationship between alveolar–capillary barrier dysfunction and the severity of cardiac hemodynamic impairment. Further, the relative increase in SPB after exercise was greater in patients with more advanced symptoms of HF, as reflected by a higher NYHA functional class. These observations are consistent with the pioneering study by De Pasquale et al. [7], which demonstrated that circulating SPB levels were elevated in patients with chronic HF in comparison with healthy controls and increased with worsening NYHA class. Moreover, in their study, SPB levels showed similar trends to changes in NT-pro-BNP levels in response to diuretic treatment modifications. Similar conclusions were later reported in a review by Banfi et al. [3], emphasizing that circulating SPB reflects the pulmonary consequences of cardiac dysfunction and may serve as a marker of alveolar–capillary barrier damage in HF.

Interestingly, despite the association between SPB dynamics and indices of HF severity and exercise capacity, we did not observe significant correlations between SPB concentrations (at rest, after exercise, or expressed as relative change) and invasive hemodynamic parameters such as systolic pulmonary artery pressure or pulmonary vascular resistance measured during right-heart catheterization. This finding suggests that the release of SPB into the circulation may not be determined solely by resting pulmonary hemodynamic load. As previously mentioned, previous studies have indicated that pulmonary involvement in HF is not only exclusively related to pulmonary artery pressure but also reflects structural and functional alterations of the alveolar–capillary membrane, including interstitial fibrosis, endothelial dysfunction, and remodeling of the extracellular matrix [5]. Such changes may impair membrane integrity and increase its susceptibility to transient mechanical stress even when resting pulmonary pressures are not markedly elevated. Moreover, right-heart catheterization reflects pulmonary hemodynamics under resting conditions, whereas the integrity of the alveolar–capillary barrier may be challenged primarily during dynamic states associated with increased pulmonary blood flow and pressure, such as physical exertion. Therefore, circulating SPB may capture aspects of pulmonary microstructural dysfunction that are not directly reflected by conventional hemodynamic measurements obtained at rest.

The potential influence of beta-blocker therapy on SPB dynamics deserves consideration, particularly because beta-adrenergic pathways are involved in the regulation of alveolar fluid clearance and surfactant homeostasis [1]. Experimental data indicate that beta-adrenergic stimulation may modulate surfactant synthesis and secretion by type II alveolar cells, although this effect appears to depend on the developmental and physiological conditions of the lung [16]. In addition, beta2-receptor-dependent mechanisms contribute to alveolar fluid removal through regulation of epithelial sodium transport and Na+/K+-ATPase activity [1]. Therefore, non-selective beta-blockade could theoretically influence alveolar–capillary function and pulmonary diffusion capacity. As reviewed by Banfi et al. [1], previous studies suggest that carvedilol, a non-selective beta1/beta2-blocker, may impair the membrane component of lung diffusion, whereas this effect was not observed with beta1-selective agents such as bisoprolol. In the present study, however, neither beta-blocker selectivity nor achievement of the target beta-blocker dose was associated with resting SPB, post-exercise SPB, relative SPB change, or CPET-derived indices of exercise capacity. These findings suggest that the observed exercise-induced SPB response was unlikely to be explained by differences in beta-blocker therapy and may rather reflect exercise-related pulmonary vascular stress and alveolar–capillary barrier vulnerability in advanced heart failure. 

Sildenafil use also requires comment, as it is not recommended as routine therapy for HF. In our cohort, sildenafil was used in selected patients with pulmonary hypertension during advanced HF evaluation rather than as standard HF therapy. Bussotti et al. [17] have suggested that sildenafil may improve pulmonary vascular loads, exercise capacities, and alveolar–capillary function in selected patients with HF, probably through enhancement of nitric oxide–cyclic guanosine monophosphate signaling. In particular, they reported that sildenafil improved lung diffusion and attenuated physiological indices of exercise-induced alveolar edema formation, as reflected by changes in the ratio of alveolar–capillary membrane diffusion capacity to pulmonary capillary blood volume (DM/VC) [17]. In addition, the meta-analysis by Zhuang et al. [18] suggested that sildenafil may improve exercise capacities in patients with HF, including peak VO_2_, anaerobic thresholds, and the VE/VCO_2_ slope. From a pulmonary hypertension perspective, Jiang et al. [19] reported that sildenafil improved pulmonary hemodynamics and CPET-derived parameters in patients with pulmonary hypertension due to left heart disease and HF with reduced ejection fraction, which is relevant to our cohort of advanced HF patients undergoing transplant evaluation. However, in the present cohort, sildenafil treatment was not associated with resting SPB, post-exercise SPB, relative SPB change, or CPET-derived indices. Therefore, sildenafil use is unlikely to have substantially influenced the observed SPB dynamics, although this exploratory analysis should be interpreted cautiously because sildenafil was used only in a small subgroup of patients.

Despite these associations with disease severity and exercise physiology, SPB levels did not predict clinical outcomes in our cohort. This finding differs from some previous studies reporting a prognostic role of surfactant proteins in HF. For example, Magrì et al. [8] demonstrated that circulating immature forms of SPB were independently associated with hospitalization for worsening HF and cardiovascular mortality in patients with stable systolic HF. Similarly, an earlier work by De Pasquale et al. [7] showed that higher plasma SPB concentrations were associated with an increased risk of HF hospitalization. Several factors may explain this discrepancy. First, the type of surfactant protein analyzed may be important. In the study by Magrì et al. [8], the immature form of SPB was measured, which may more directly reflect structural damage of the alveolar–capillary membrane. Biologically, immature SPB is located within intracellular organelles and is released into the circulation mainly in the setting of substantial pneumocyte injury, whereas the mature SPB form assessed in our study may cross the alveolar–capillary membrane during reversible mechanical stretch related to elevated pulmonary microvascular pressure [8]. This interpretation is further supported by the study by Gargiulo et al. [4], which demonstrated that among various surfactant-derived proteins, immature SPB showed the strongest association with impaired lung diffusion capacity, whereas mature SPB did not correlate with key indices of pulmonary dysfunction or HF severity. These findings suggest that different SPB isoforms reflect distinct pathophysiological processes, with immature forms being more closely related to structural damage of the alveolar–capillary membrane. Consequently, immature SPB may represent a marker of irreversible alveolar epithelial damage, while mature SPB may primarily reflect transient permeability changes in the barrier. Consistent with this interpretation, prognostic associations have also been reported for other surfactant proteins. Circulating surfactant D (SPD) levels have been linked with cardiovascular morbidity and mortality in population cohorts [20], and recent large-scale proteomic studies in HF identified surfactant C (SPC) among proteins associated with adverse outcomes [21], suggesting that different surfactant isoforms may capture distinct aspects of pulmonary involvement in cardiovascular disease. Second, differences in the studied populations may also have influenced the results. Our cohort consisted of patients with advanced HF referred for heart transplantation evaluation, in whom disease severity was already markedly increased. In addition, a substantial proportion of patients already presented features of pulmonary hypertension, indicating advanced and likely chronic involvement of the pulmonary circulation. In such a population, pulmonary barrier dysfunction may be relatively homogeneous, which could limit the ability of SPB levels to further discriminate prognosis. Finally, the study by De Pasquale et al. [7] evaluated a less stringent endpoint focused mainly on HF hospitalization, which may be more sensitive to markers of pulmonary congestion than the harder composite outcomes analyzed in the present study.

### 3.1. Study Limitations

Several limitations should be acknowledged. This was a single-center study with a relatively small sample size, which may limit the generalizability of the results. The population consisted of patients with advanced HF undergoing evaluation for heart transplantation, and therefore, the findings may not apply to patients with milder disease. Only circulating SPB levels were measured, whereas immature SPB isoforms or other surfactant proteins such as SPC or SPD might provide additional prognostic information. Moreover, the ELISA used in this study detects both mature and precursor forms of SPB but does not allow their differentiation; therefore, the results reflect total circulating SPB rather than specific isoforms. This approach may capture a broader spectrum of SPB release, including transient exercise-related increases related to alveolar–capillary barrier permeability. However, by combining mature SPB with immature forms that may more specifically reflect severe or irreversible pneumocyte injury, total SPB measurement may also dilute the prognostic signal that could be obtained from isoform-specific assessment.

The observational design precludes establishing causal relationships between exercise-induced pulmonary stress and surfactant leakage.

Another limitation is the lack of a control group. However, the present study was specifically designed to investigate exercise-induced changes in circulating SPB within a clinically relevant population of patients with advanced HF referred for heart transplantation evaluation rather than to compare absolute biomarker levels between patients and healthy individuals. Previous studies have consistently demonstrated that resting circulating surfactant protein levels are elevated in patients with heart failure compared with healthy controls [4,7,22], indicating lower baseline SPB concentrations in individuals without cardiac disease. Furthermore, exercise-induced increases in circulating SPB have been observed in patients with exercise-induced left ventricular dysfunction, whereas no such response was detected in individuals without cardiac impairment [23]. However, these studies were performed in different clinical settings and do not directly address SPB dynamics during standardized cardiopulmonary exercise testing in patients with advanced HF. To our knowledge, no studies have directly compared SPB responses to CPET between patients with HF and healthy controls.

Taken together, these findings provide a strong physiological and clinical framework supporting the interpretation of our results. Therefore, although the absence of a control group limits direct comparisons, it does not diminish the validity of the observed exercise-related SPB dynamics within this well-defined heart failure cohort. 

In addition, SPB was measured only at two time points—at rest and shortly after exercise—which precludes detailed assessment of the full temporal dynamics of SPB release and clearance in the circulation.

Detailed causes of death were not systematically available for all patients and, therefore, could not be analyzed reliably. In addition, the relatively small sample size and limited number of clinical events restrict the statistical power of the prognostic analyses and should be interpreted with caution. Therefore, the present findings should be considered exploratory and hypothesis-generating rather than definitive.

### 3.2. Clinical Implications

The main significance of this study is mechanistic and pathophysiological rather than purely prognostic. The novelty of our work lies not in demonstrating SPB as a survival predictor but in assessing the dynamic SPB response to a standardized physiological stressor in a uniquely characterized cohort of patients with advanced HF referred for heart transplant evaluation. This is not a general HF population but a clinically important group in whom exercise limitation, pulmonary vascular load, and pulmonary barrier dysfunction may all contribute to disease severity and therapeutic decision-making.

A major strength of this study is the combination of paired pre- and post-exercise SPB measurements with CPET data, invasive resting hemodynamic assessment, and follow-up outcomes. To our knowledge, such an integrated evaluation of exercise-induced SPB response in advanced HF in relation to both ventilatory exercise parameters and invasive pulmonary hemodynamics has not been previously reported. The observed increase in SPB after exercise, together with its association with disease severity and impaired exercise capacity, suggests that circulating SPB may provide insight into the pulmonary component of HF pathophysiology. Importantly, the lack of association with resting systolic pulmonary artery pressure and pulmonary vascular resistance further suggests that this exercise-related pulmonary barrier response may not be captured by resting invasive hemodynamic measurements alone. Specifically, these findings support the concept that acute exertion can transiently aggravate alveolar–capillary barrier stress in advanced HF and that this dynamic response is linked to functional limitations.

Importantly, markers of alveolar–capillary membrane dysfunction appear to be modifiable with effective HF therapy. A previous study has shown that treatment with sacubitril/valsartan is associated with a reduction in circulating surfactant proteins such as proSPB and SPD, accompanied by improvements in cardiac function and pulmonary parameters [24], suggesting partial reversibility of pulmonary barrier dysfunction in HF.

These observations indicate that surfactant-derived biomarkers may complement traditional cardiac markers by providing additional information on lung–heart interactions and on the pulmonary response to therapy in patients with HF.

## 4. Materials and Methods

### 4.1. Study Population and Design

This prospective study was performed at a tertiary cardiology center. The analyzed cohort consisted of 51 patients with chronic HF referred for evaluation for heart transplantation. The study group and general study procedures have been described previously [10]; briefly, patients were 36–66 years old (mean 55.6 ± 7.5 years), 9 (16%) were women, and all had advanced systolic HF with NYHA class II–III symptoms and left ventricular ejection fractions below 35% (mean 21.7 ± 5.4%). Detailed baseline characteristics are provided in Table 2 [10]. Patients were excluded in the presence of catecholamine treatment, contraindications to cardiopulmonary exercise testing, recent lower respiratory tract infection, or severe ventilatory impairment defined as FEV1 < 50%. All participants received guideline-directed medical therapy applicable at the time of enrollment.

As part of the diagnostic work-up to assess exercise capacity, all patients underwent symptom-limited cardiopulmonary exercise testing on a bicycle ergometer (Lode Medical Technology, Groningen, the Netherlands) under physician supervision using the same protocol we described in our earlier reports [10,11]. Before each test, the gas analyzer and flow sensor were calibrated according to the manufacturer’s recommendations. Exercise began with 3 min of unloaded pedaling, followed by a graded workload increase of 10 W/min. Continuous electrocardiographic monitoring was performed throughout the test, and blood pressure and symptoms were assessed repeatedly during exercise and recovery. All tests were terminated at the patient’s request because of limiting symptoms, mainly dyspnea or fatigue. Oxygen consumption (VO_2_), carbon dioxide output (VCO_2_), and ventilatory parameters were measured using the MetaLyzer 3B-R2 system (Cortex, Leipzig, Germany). Peak VO_2_ was defined as the highest 30 s averaged value recorded immediately before exercise cessation, and age- and sex-adjusted peak VO_2_ was calculated automatically by the system’s software. The respiratory exchange ratio was calculated as VCO_2_/VO_2_. Ventilatory efficiency was assessed by the VE/VCO_2_ slope, defined as the relationship between minute ventilation and carbon dioxide production. The values of the anaerobic thresholds were obtained from the CPET report generated by the system’s software. After exercise, all patients completed at least 2 min of unloaded recovery on the bicycle. The CPET results are summarized in Table 2 [10].

All patients underwent invasive right heart catheterization as part of the diagnostic evaluation. Pulmonary vascular resistance (PVR) was calculated using cardiac output derived according to the Fick principle.

The study endpoint was defined as death, urgent heart transplantation, or urgent left ventricular assist device (LVAD) implantation. Patients were followed for a mean of 23.5 ± 12.6 months (range 2–44 months), and follow-up was censored at the date of death, any heart transplantation, or any LVAD implantation.

This study’s workflow is presented schematically in the flowchart (Supplementary Figure S3).

### 4.2. Blood Sampling and Laboratory Methods

Venous blood was collected at rest and subsequently during the post-exercise recovery period, 10–15 min after the end of the cardiopulmonary exercise test. Plasma was separated by centrifugation, immediately frozen, and stored at −80 °C until further analysis.

SPB was determined by the enzyme-linked immunosorbent assay (ELISA) using a commercial kit (ELISA, SEB622Hu, Wuhan USCN Business Co., Ltd., Wuhan, China). A microplate pre-coated with anti-surfactant protein B (SPB) antibodies was used. The assay detects both mature (~6–8 kDa) and precursor (immature, ~18 kDa) forms of SPB, but it does not allow discrimination between these molecular forms.

A series of SPB standards was prepared by dissolving the lyophilized standard in 1.0 mL of standard diluent, followed by incubation at room temperature (RT) for 10 min to ensure complete dissolution. A two-fold serial dilution was performed in seven wells, starting with an initial concentration of 100 ng/mL, followed by 50 ng/mL, 25 ng/mL, 12.5 ng/mL, 6.25 ng/mL, 3.12 ng/mL, and 1.56 ng/mL. A blank well containing diluent only was included as a negative control.

For the sample and standard application, 100 μL of each plasma sample and standard dilution was added to the appropriate wells of the microplate. The plate was sealed and incubated at 37 °C for 2 h. After incubation, the liquid was carefully removed without washing.

Next, 100 μL of Detection Solution A was added to each well, and the plate was incubated at 37 °C for 1 h. The solution was discarded, and each well was washed three times with 350 μL of the prepared Wash Buffer (1×). Then, 100 μL of Detection Solution B was added to each well, followed by incubation at 37 °C for 30 min. The wash step was then repeated five times to ensure the removal of unbound reagents.

After washing, 90 μL of the TMB substrate solution was added to each well, and the plate was incubated at 37 °C for 15–25 min in the dark. The reaction was stopped by adding 50 μL of stop solution to each well, resulting in a color change to yellow. Absorbance was measured at 450 nm within 10 min using a microplate reader (LEDETECT 96 microplate reader, MikroWin software, version 4.41, Biomed Dr. Wieser GmbH, Salzburg, Austria).

The optical density (OD) values of the standards and plasma samples were used to construct a standard curve: y = 0.5016x + 0.093, R^2^ = 0.9875. The mean OD values from duplicate measurements were plotted against the corresponding standard concentrations to quantify SPB in the plasma samples.

### 4.3. Statistical Methods

To normalize the exercise response to baseline levels, the relative change in surfactant protein B (SPB) was calculated as (SPB_post-exercise_ − SPB_at rest_)/SPB_at rest_.

Continuous variables are presented as mean ± standard deviation for normally distributed data or as median [interquartile range] for non-normally distributed data, and categorical variables as percentages. Normality of data distribution was assessed with the Shapiro–Wilk test. Comparisons between paired measurements were performed using the paired Student’s *t*-test or the Wilcoxon signed-rank test, as appropriate. Differences between independent groups were analyzed using the unpaired Student’s *t*-test or the Mann–Whitney U test, as appropriate. Correlations between continuous variables were evaluated using Spearman’s rank correlation analysis. Univariable Cox proportional hazards models were used to assess predictors of survival. Event-free survival was analyzed using Kaplan–Meier survival curves. Patients were stratified according to the median value of the relative change in SPB. Differences between survival curves were assessed using the log-rank test. Statistical analyses were performed with Statistica 12.0 software. A two-tailed *p* value < 0.05 was considered statistically significant.

## 5. Conclusions

In patients with advanced heart failure, circulating surfactant protein B increases after cardiopulmonary exercise testing, and its dynamics are associated with markers of disease severity and impaired exercise capacity. These findings support the concept that physical exertion may transiently aggravate alveolar–capillary barrier dysfunction in heart failure. However, neither baseline nor exercise-induced changes in SPB were associated with clinical outcomes or with invasive hemodynamic parameters of the pulmonary circulation, including pulmonary artery pressure and pulmonary vascular resistance, in this cohort. Circulating SPB may therefore reflect dynamic pulmonary stress and the functional interaction between the heart and lungs in heart failure. Importantly, the magnitude of the exercise-induced SPB response was not associated with prognosis in this cohort; however, this finding should be interpreted with caution given the limited sample size and number of events, and it warrants further investigation in larger studies.

## Figures and Tables

**Table 1:** Correlation between surfactant B plasma concentrations and invasive pulmonary hemodynamic parameters.

	N	R Spearman	*p* Value
SPB at rest & sPAP	51	0.06	0.67
SPB at rest & PVR	51	0.02	0.91
SPB after CPET & sPAP	51	0.06	0.66
SPB after CPET & PVR	51	0.05	0.71
SPB increase & sPAP	51	0.06	0.67
SPB increase & PVR	51	0.13	0.35

CPET—Cardiopulmonary exercise test; PVR—pulmonary vascular resistance; sPAP—systolic pulmonary artery pressure; SPB—surfactant B; SPB increase—relative change in surfactant protein B (SPB) was calculated as (SPB_post-exercise_ − SPB_at rest_)/SPB_at rest_.

**Table 2:** Clinical characteristics of the study population [10].

	Study Population (N = 51)
Clinical characteristics	
Age, years	55.6 ± 7.5
Gender, % male	43 (84.3%)
Ischemic etiology of HF	31 (60.8%)
Atrial fibrillation	24 (47.1%)
Hypertension	17 (33.3%)
Diabetes mellitus	20 (39.2%)
COPD	5 (9.8%)
ICD/CRT-D	30 (58.8%)/14 (27.5%)
II NYHA class, patients (%)	22 (43.1%)
III NYHA class, patients (%)	29 (56.9%)
Echocardiographic parameters	
LVEF [%]	21.7 ± 5.4
LVEDD [mm]	73.5 ± 9.6
TAPSE [mm]	17.0 ± 3.3
Mitral regurgitation *, patients (%)	37 (72.5%)
Tricuspid regurgitation *, patients (%)	24 (47.1%)
Laboratory test results	
NT-pro-BNP [pg/mL], median (interquartile range)	2604 [1185–4827]
eGFR, mL/min/1.73 m^2^	59.3 ± 16.1
bilirubin [mmol/L]	19.4 ± 9.1
Medication	
Beta-blockers [n, %]	51 (100.0)
Target beta-blocker dose achieved [n, %]	19 (37.3)
Selective beta-blockers [n,%]	30 (58.8)
ACEI/ARB/ARNI [n, %]	51 (100.0)
Spironolactone/eplerenone	50 (98.0)
Loop diuretics [n, %]	50 (98.0)
More than one diuretic administered (except for MRA) [n, %]	26 (51.0)
Sildenafil, patients [n, %] ^#^	11 (21.6)
Allopurinol, patients [n, %]	21 (41.2)
Cardiopulmonary exercise test results	
pVO2 [mL/kg/min]	10.5 ± 2.9
pVO2 adjusted for sex and age adjusted pVO2 [%]	38.6 ± 12.6
RER at peak exhaustion	1.1 ± 0.1
VE/VCO_2_ slope	43.9 ± 12.3
AT [mL/kg/min]	8.1 ± 2.6
Right heart catheterization	
sPAP [mmHg]	54.3 ± 18.8
PVR [Wood units]	1.7 [1.7–3.8]

Data are presented as mean ± standard deviation, median [interquartile range], or as number (percentages). ACEI = Angiotensin-converting enzyme inhibitor; ARB = angiotensin receptor blocker; ARNI = angiotensin receptor–neprilysin inhibitor; AT—anaerobic threshold; COPD = chronic obstructive pulmonary disease; CRT-D = cardiac resynchronization therapy defibrillator; eGFR = estimated glomerular filtration rate; HF = heart failure; ICD = implantable cardioverter defibrillator; LVEDD = left ventricular end-diastolic dimension; LVEF = left ventricular ejection fraction; NYHA = New York Heart Association; MRA = mineralocorticoid receptor antagonist; NT-pro-BNP = N-terminal prohormone of brain natriuretic peptide; pVO2 = peak oxygen uptake; PVR = pulmonary vascular resistance; RER = respiratory exchange ratio; sPAP = systolic pulmonary artery pressure; TAPSE = tricuspid annular plane systolic excursion; VE/VCO_2_ = minute ventilation/carbon dioxide production. * Mitral or tricuspid regurgitation at least moderate. ^#^ Sildenafil was used in selected patients with pulmonary hypertension during advanced HF/transplant evaluation and was not prescribed as heart failure therapy per se.

## Data Availability

The data presented in this study are available on request from the corresponding author due to privacy restrictions related to patients’ data.

## Supplementary Materials

The following supporting information can be downloaded at https://www.mdpi.com/article/10.3390/ijms27146461/s1.

## Abbreviations

The following abbreviations are used in this manuscript:

ACEI	Angiotensin-converting enzyme inhibitor;
ARB	Angiotensin receptor blocker;
ARNI	Angiotensin receptor–neprilysin inhibitor;
AT	Anaerobic threshold;
COPD	Chronic obstructive pulmonary disease;
CPET	Cardiopulmonary exercise test;
CRT-D	Cardiac resynchronization therapy defibrillator;
eGFR	Estimated glomerular filtration rate;
HF	Heart failure;
ICD	Implantable cardioverter defibrillator;
LVEDD	Left ventricular end-diastolic dimension;
LVEF	Left ventricular ejection fraction;
NYHA	New York Heart Association;
MRA	Mineralocorticoid receptor antagonist;
NT-pro-BNP	N-terminal prohormone of brain natriuretic peptide;
pVO2	Peak oxygen uptake;
PVR	Pulmonary vascular resistance;
RER	Respiratory exchange ratio;
sPAP	Systolic pulmonary artery pressure;
SPB	Surfactant B;
SPC	Surfactant C;
SPD	Surfactant D;
TAPSE	Tricuspid annular plane systolic excursion;
VE/VCO_2_	Minute ventilation/carbon dioxide production.
