# Supplementary material for: Exercise-Induced Plasma Surfactant Protein B Response in Advanced Heart Failure: Relation to Exercise Limitation, Resting Invasive Hemodynamics, and Clinical Outcomes
Source: Int J Mol Sci. 2026 Jul 21;27(14):6461. doi: 10.3390/ijms27146461 (PMC13409963; doi:10.3390/ijms27146461)
Supplement: Supplementary file 1 [file ijms-27-06461-s001.zip › ijms-4412615-supplementary.pdf]

Supplementary materials

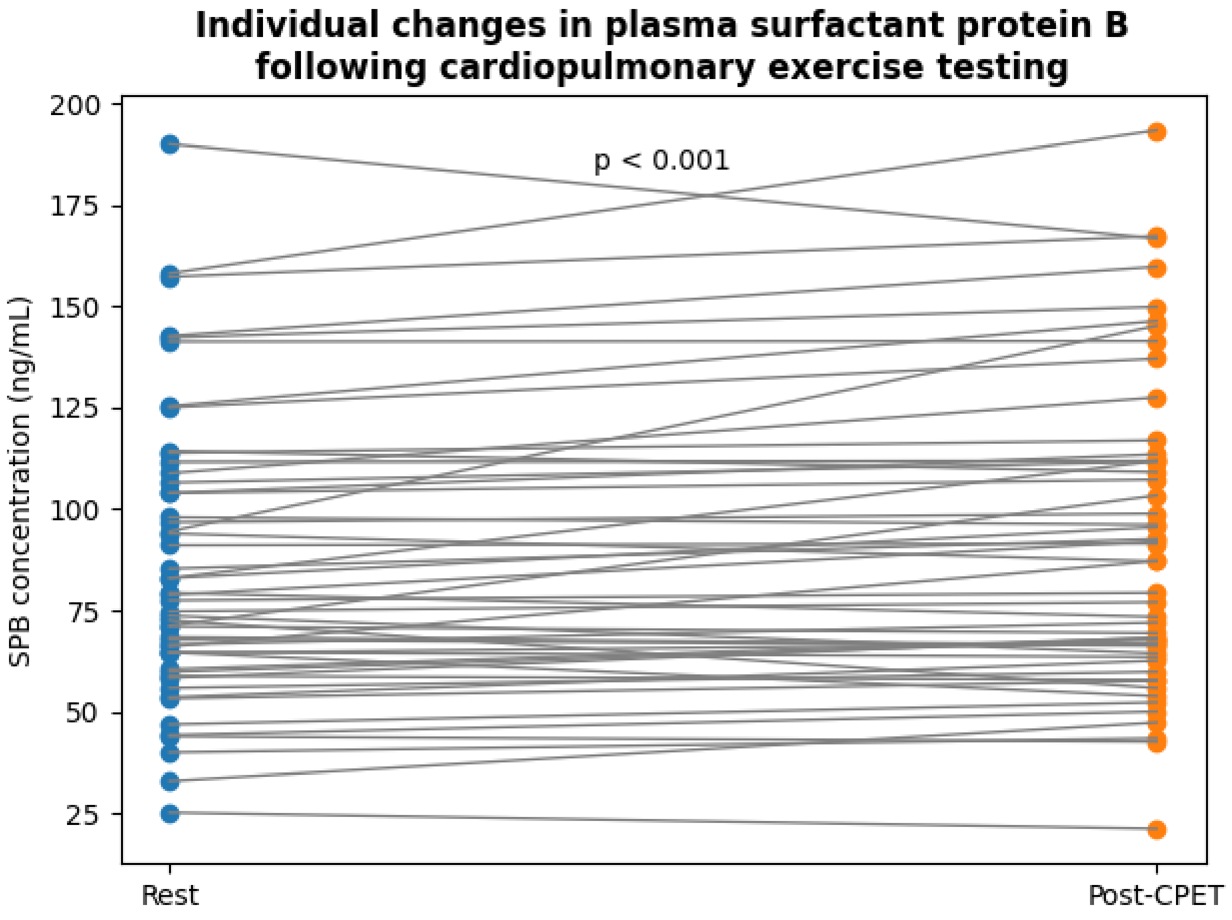

Supplementary Figure S1A . Individual changes in plasma surfactant protein B (SPB) concentrations before and after cardiopulmonary exercise testing in 51 patients. Each line represents a single patient.

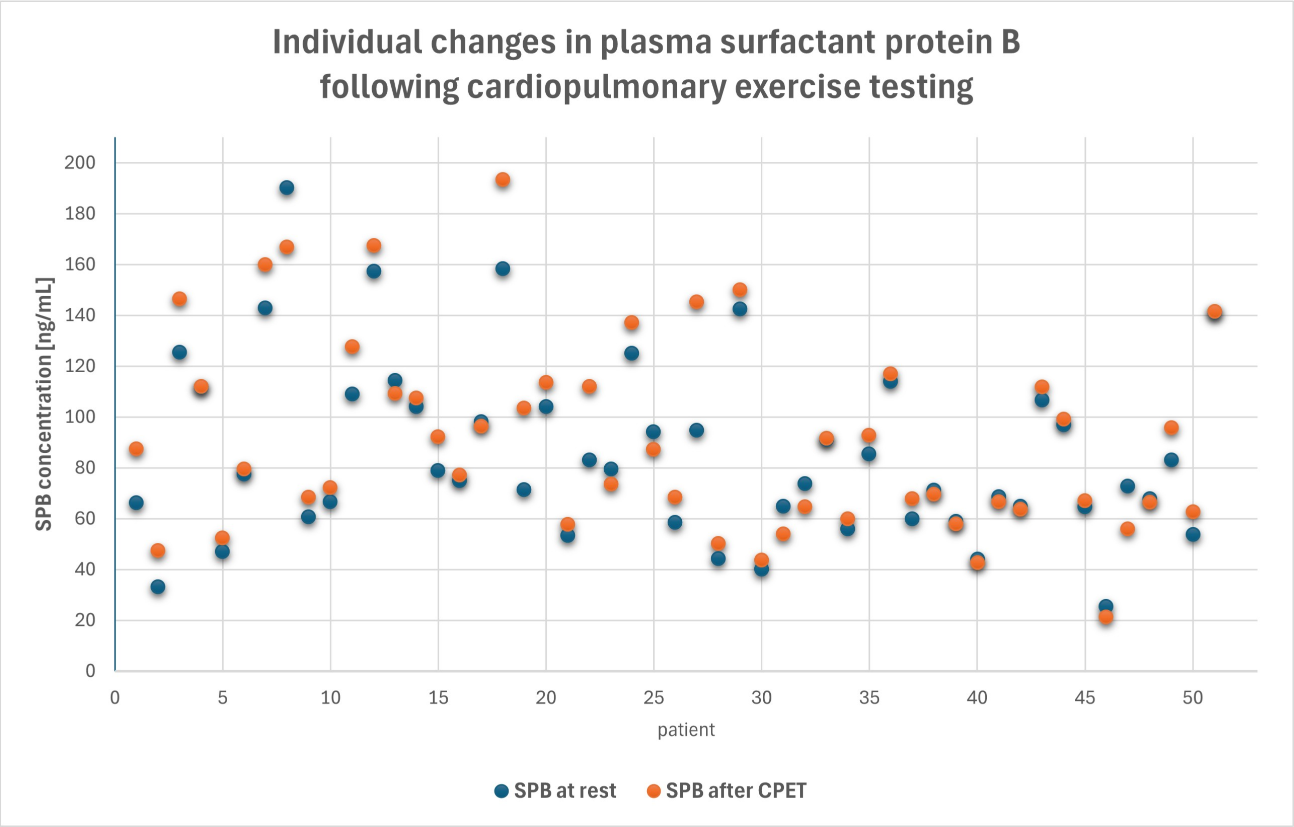

Supplementary Figure S1B. Plasma surfactant protein B (SPB) concentrations at rest and after cardiopulmonary exercise testing (CPET) in individual patients (n = 51). Each point represents a single measurement. SPB levels increased significantly after CPET (*$p < 0.001$).

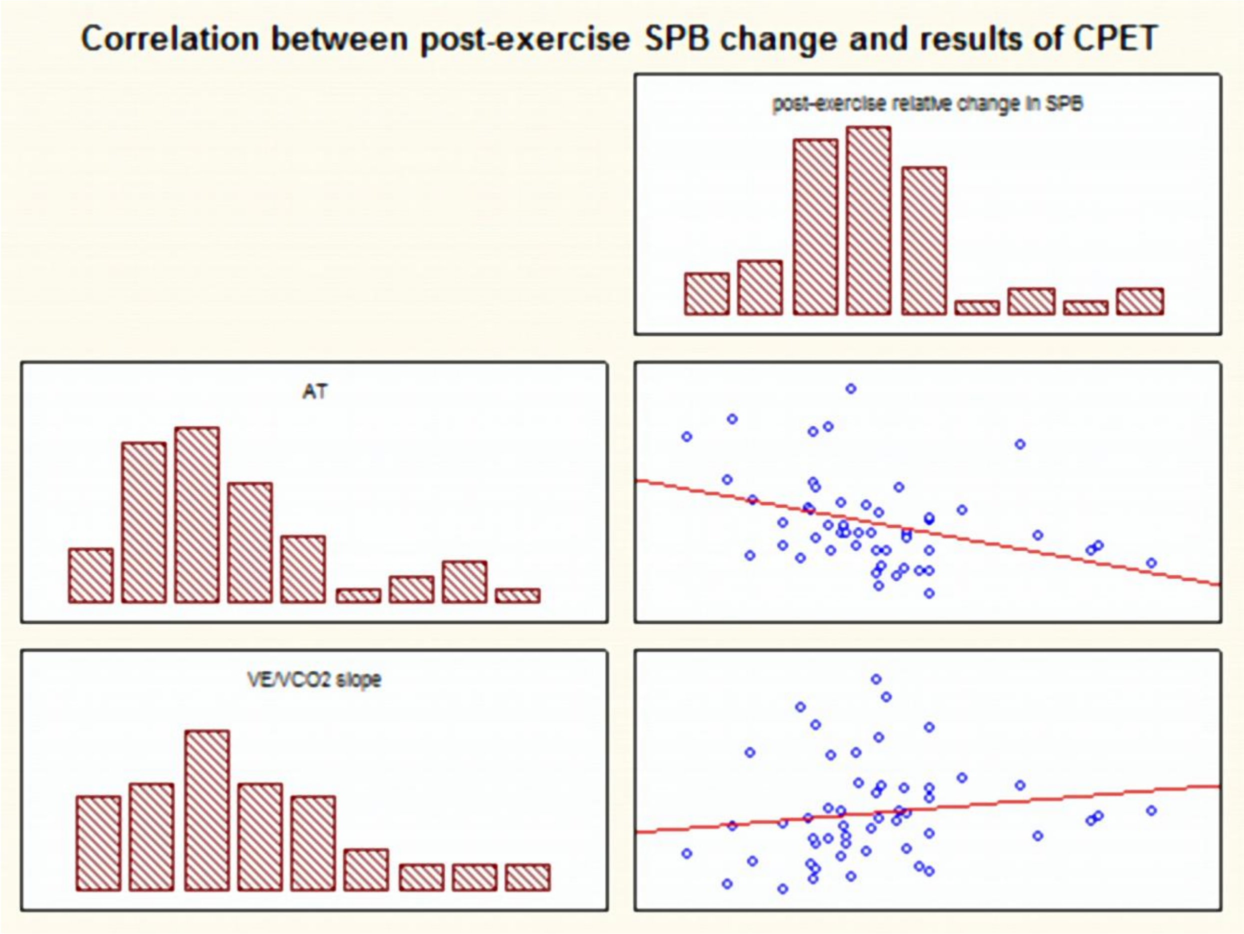

Supplementary Figure S2. Correlation between the relative change in plasma surfactant protein B (SPB) after cardiopulmonary exercise testing (CPET) and indices of exercise capacity. Scatter plots illustrate the relationships between post-exercise SPB change and anaerobic threshold (AT) and the VE/VCO_2 slope, with corresponding linear regression lines. Histograms show the distribution of analyzed variables. Line shown for visualization purposes

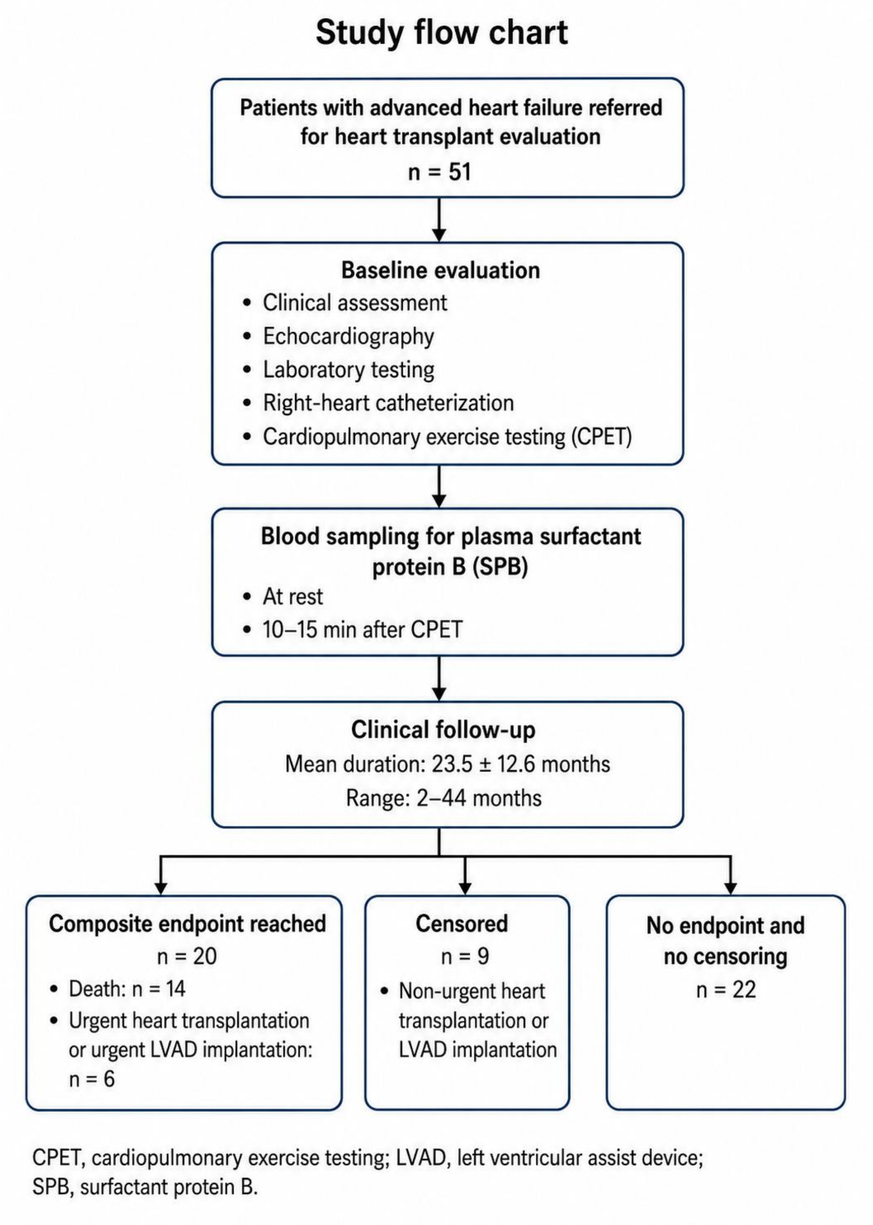

Supplementary Figure S3. Study flow chart. Fifty-one patients with advanced heart failure referred for heart transplant evaluation underwent baseline evaluation, including clinical assessment, echocardiography, laboratory testing, right-heart catheterization, and cardiopulmonary exercise testing (CPET), with plasma surfactant protein B (SPB) measured at rest and 10–15 min after exercise. During a mean follow-up of 23.5 ± 12.6 months, 20 patients reached the composite endpoint, including 14 deaths and 6 urgent heart transplantations or urgent left ventricular assist device (LVAD) implantations; 9 patients were censored at the time of non-urgent heart transplantation or LVAD implantation, and 22 remained free of endpoint and censoring.
